# EndoSheath use in flexible cystoscopy: a prospective evaluation of >1000 cases

**DOI:** 10.1111/bju.16578

**Published:** 2024-11-12

**Authors:** Lara Ratcliffe, Brian Birch

**Affiliations:** ^1^ School of Medicine University of Southampton Southampton UK; ^2^ University Hospital Southampton Southampton UK

**Keywords:** flexible cystoscopy, EndoSheath, urinary tract infection, tolerability, discomfort

## Abstract

**Objectives:**

To investigate patient tolerability and safety (using urinary tract infection (UTI) as a proxy measure) following EndoSheath‐assisted flexible cystoscopy (eFC). EndoSheaths are single‐use, disposable sheaths used in FC. They reduce cystoscope turnaround times as complicated, time‐consuming and costly sterilisation is no longer necessary. This reduces patient waiting times as cystoscope idle time, the most common rate limiting step, is reduced.

**Patients and Methods:**

All adult patients undergoing eFC over a 26‐month period at a single institution were evaluated prospectively. Post‐eFC, participants rated discomfort on a visual analogue scale (discomfort 1 = none, 2–4 = mild, 5–7 = moderate, 8–10 = severe). The diagnosis of UTI was broad and based on any one of patient self‐report, positive urine culture or antibiotic prescription within 30 days of eFC. Data were analysed using chi‐squared testing (*P* < 0.05, two‐tailed).

**Results:**

Of the 1091 eFCs analysed, 33.2% and 48.2% of were ranked as causing no or mild discomfort, respectively, with just 3.1% ranked severe. Discomfort was greater in younger participants but similar between sexes. Overall, post‐eFC UTI incidence was 13.3%, with rates higher in females (18.1%) than males (11.2%). Participants aged ≥65 years reported a higher UTI rate (15.4%) than those aged <65 years (8.8%). No participants developed urosepsis.

**Conclusion:**

This large, prospective, unselected, real‐world study reports that eFC is well tolerated. UTI was higher in females than males, and in those aged ≥65 years, in line with other studies using the same broad based diagnostic criteria for UTI. The conclusion is that eFC is both well tolerated and safe.

**Abbreviations**
CFU: colony‐forming units
(e)FC: (EndoSheath‐assisted) flexible cystoscopy
MSU: mid‐stream urine
VAS: visual analogue scale

## Introduction

Flexible cystoscopy (FC) is the most commonly used procedure in urology, and the ‘gold standard’ for the diagnosis and surveillance of bladder cancer [1, 2]. Cystoscopes need sterilising between patients, which is both costly and time consuming [3]. An EndoSheath provides a barrier between patients and the cystoscope and can be simply removed and replaced between procedures [1, 4].

EndoSheath‐assisted FC (eFC) uses a disposable sheath, a technique used by relatively few endoscopy centres in the UK. It negates the need for full sterilisation of the cystoscope between patients [1]. This paper evaluates its tolerability and safety in a prospective real‐world study of >1000 patients.

It is hoped that by confirming that eFC is tolerable and safe, this research will commend the technology to other centres. This has the potential to allow more patients to be scoped on an operating list and at reduced operating cost due to time savings for cystoscope sterilisation. This is important given increased patient waiting lists as the NHS recovers from COVID‐19 [5].

Flexible cystoscopy has been in use since 1973 as an alternative to rigid cystoscopy [6]. It is associated with lower pain compared to rigid cystoscopy and obviates the need for general anaesthesia [7, 8].

Flexible cystoscopy is the most commonly performed urological intervention and serves as a diagnostic, surveillance and treatment tool [1]. According to the European Association of Urology, cystoscopy it is necessary for the diagnosis of bladder cancer [9]. Other indications for FC include the investigation (and possible treatment) of recurrent UTIs, haematuria, LUTS, bladder stones and bladder cancer monitoring or treatment.

In 2017/2018 the number of FCs performed in the UK each year was estimated to be between ~110 000 and ~257 565, at a NHS cost of £55.39 million [10]. Globally, >4 million procedures are performed annually [11].

An EndoSheath is a single‐use, disposable sheath placed over the flexible cystoscope. In general use, EndoSheaths decrease endoscope reprocessing (cleaning and sterilisation) times ~nine‐fold and ‘idle time’ (often the rate‐limiting step), improving list usage in studies of EndoSheath‐assisted endoscopy [12].

In urology, EndoSheaths circumvent the need for extensive and time‐consuming sterilisation. The cystoscope is simply wiped with 70% ethanol and then dried [3, 4, 13, 14] between patients, as per National Institute for Health and Care Excellence (NICE) guidelines. Total set‐up and reprocessing time when using an EndoSheath varies between ~2 and 11 min [1, 13, 15], significantly faster than for a standard cystoscope (30–64 min [13, 15]). The difference is mainly due to the disinfection step taking ~0.5–1.5 min [4, 13] for eFC, but 14–45 min for a single reusable scope. Whilst this aids list efficiency other factors need consideration such as patient and room preparation etc. However, there are still cost savings to be made by the reduction in labour and time. In addition, high level disinfection for reusable cystoscopes is costly and EndoSheath use circumvents this.

Studies report no significant difference in image quality when using an EndoSheath [1, 15].

In standard cystoscopy (no EndoSheath), asymptomatic bacteriuria has been observed in 24% of patients and *Escherichia coli* in 41% cultures post‐cystoscopy [16], whilst UTI rates vary from 1.9% to 4.5% [16] to 7.5% [17], with just 1.94% febrile UTIs [16].

The primary aim of this study was to investigate patient tolerability of eFC. A secondary aim was an assessment of post‐eFC UTI rates.

## Patients and Methods

### Clinical Procedures

All adult patients undergoing eFC at a single institution (University Hospital Southampton, UK) over a 26‐month period participated in this prospective evaluation.

Participants were consented for routine eFC on the day of their procedure, following which they provided, if possible, a urine sample for dipstick urine analysis.

The eFCs were conducted using the VISION SCIENCES CST‐5000 PrimeSight™ cystoscope (charge‐coupled device‐based, high‐resolution) with an EndoSheath® protective barrier [18].

Immediately following eFC, participants completed an assessment of procedural tolerability by rating discomfort on a 10‐point categorical visual analogue scale (VAS) and were given instructions related to a second survey concerning the occurrence of UTI post‐eFC.

In the 4 weeks following eFC, participants were asked to complete and return the second survey asking if they experienced a post‐eFC UTI (‘Yes/No’ response). This was self‐reported, and a positive mid‐stream urine (MSU) sample was not mandatory for diagnosis. Whilst less robust than asking patients to provide an MSU sample for analysis either in the event of infection or at a given time point funding was not available to do this and a more pragmatic approach was used.

Ethical oversight was provided by the Faculty of Medicine, University of Southampton (ERGO 56520).

### Data Analysis

Demographic data, pre‐eFC urine analysis (to check for pre‐existing UTI), VAS score and post‐eFC UTI data were collected.

If the pre‐eFC dipstick urine analysis was positive for nitrites, had raised leucocyte levels and the patient complained of UTI symptoms, eFC was not performed as per institution guidelines. If the participant had a positive dipstick but was asymptomatic for UTI, eFC was permitted. If no pre‐eFC urine specimen was provided, either because the participant failed to provide a sample and/or if there was no complaint of symptoms suggestive of UTI, the participant underwent eFC but was excluded from the analysis of pre‐eFC UTI status. Whilst desirable, it was not mandatory, for patients to provide a urine sample pre‐eFC for dipstick testing.

If the second survey relating to post‐eFC UTI (see Data S1) was not returned, CHARTS (an electronic database system) was interrogated to determine if the participant had experienced a UTI in the month following eFC: a positive MSU, a GP clinical diagnosis of UTI or prescription of antibiotics indicative of UTI, were taken as evidence of UTI even in the absence of a positive urine culture.

Participants ranked discomfort categorically (see Data S1), with results being analysed qualitatively using the ranking: 1 = no discomfort; 2–4 = mild discomfort; 5–7 = moderate discomfort; 8–10 = severe discomfort.

The eFCs for consented participants who were subsequently found to have been prescribed systemic antibiotics for any reason in the week prior to eFC were excluded from all analyses.

Data were analysed by procedure (all) and by subgroups of sex and age (<65 years ‘younger’, ≥65 years ‘older’).

Statistical analysis was performed using chi‐squared testing, with a two‐tailed *P* < 0.05 considered statistically significant.

## Results

**Fig. 1:**
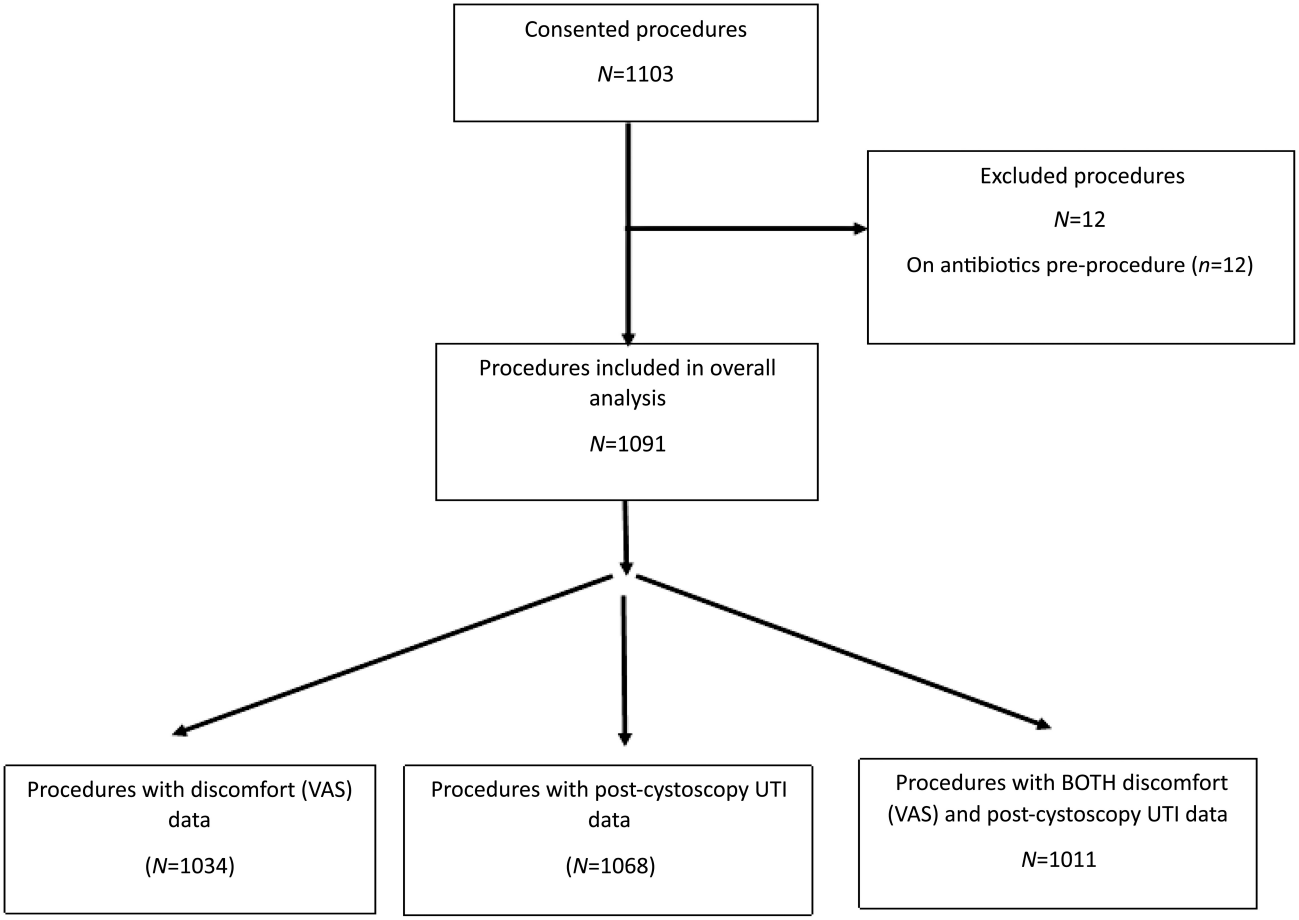
The eFCs evaluated. *N*, number of evaluable eFCs in dataset.

Over 26 consecutive months, 1103 eFCs were performed in 933 consented participants. In all, 12 eFCs were excluded from the overall analyses as antibiotics had been prescribed pre‐eFC. Therefore, the overall analysis dataset included 1091 eFCs. Data on discomfort were provided for 94.8% (1034) of eFCs and on post‐eFC UTI for 97.9% (1068). Overall, 92% (1011) of eFCs had data on both discomfort and post‐eFC UTI. Information provided in Fig. 1.

**Fig. 2:**
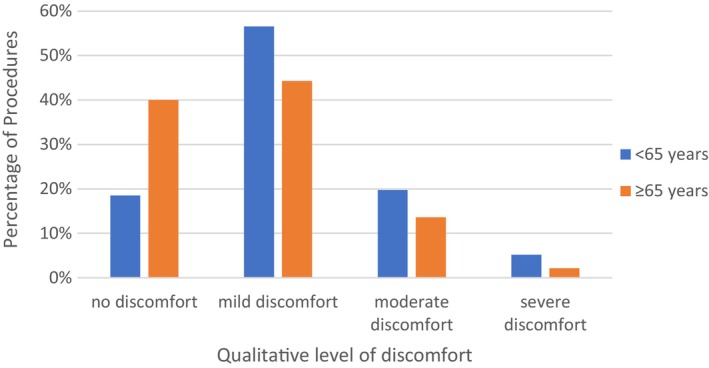
Summary of discomfort using qualitative ranking by age subgroup.

### Demographic Data Is Shown in Table 1

**Table 1:** Demographics – all eFCs and by sex and age group.

Variable	All*N* = 1091	Sex		Age	
		Male*N* = 765	Female*N* = 326	<65 years*N* = 347	≥65 years*N* = 744
Sex, *n* (%)					
Male	765 (70.1)			225 (64.8)	540 (72.6)
Female	326 (29.9)			122 (35.2)	204 (27.4)
Age (years)					
Median (SD)	71 (13.99)	71 (12.96)	69 (15.85)	55 (10.49)	76 (7.17)
Range	18–95	22–95	18–94	18–64	65–95
Mean (SE)	68.8 (0.42)	69.88 (0.47)	66.3 (0.88)	52.39 (0.56)	76.45 (0.26)
<65 years, *n* (%)	347 (31.8)	225 (29.4)	122 (37.4)		
≥65 years, *n* (%)	744 (68.2)	540 (70.6)	204 (62.6)		
Pre‐eFC positive dipstick, *n* (%)					
Yes	12 (1.1)	5 (0.7)	7 (2.1)	2 (0.6)	10 (1.3)
No	737 (67.6)	514 (67.2)	223 (68.4)	238 (68.6)	499 (67.1)
Missing*	342 (31.3)	246 (32.2)	96 (29.4)	107 (30.8)	235 (31.6)

*n*, number reporting; *N*, total number of evaluable eFCs for assessment; SD, standard deviation; SE, standard error of the mean.

* No pre‐eFC urine specimen was provided; participants reported no UTI symptoms.

The participant median (range) age was 71 (18–95) years with 70.1% of eFCs performed in males and 68.3% in those aged ≥65 years.

Dipstick urine analysis results were positive for nitrites and raised leucocytes, but patients asymptomatic for UTI, in 2.1% (seven of 326) eFCs in females and 0.7% (five of 765) eFCs in males (*P* = 0.030).

### By Age

Just over two thirds (744/1091 [68.2%]) of the eFCs were in patients aged ≥65 years. In those asymptomatic for UTI, dipstick urine analyses were positive for nitrites and raised leucocytes in 0.6% (two of 347) of eFCs in the <65 years group and 1.3% (10/744) of eFCs in the ≥65 years group (*P* = 0.258).

### By Sex

Over two thirds of all eFCs were performed in males (males: 765/1091 [70.1%]; females: 326/1091 [29.9%]). The median age of males and females was similar (males: 71 years; females: 69 years), although the proportion aged ≥65 years was higher in males (540/765 [70.6%] vs 204/326 [62.6%], *P* = 0.009).

### Tolerability and Post‐eFC UTI

#### Tolerability

Surveys on discomfort during the eFC were completed for a total of 1034 eFCs and results shown in Table 2 and Fig. 2.

**Table 2:** Summary of discomfort using qualitative ranking – all eFCs and by sex and age group.

Variable, *n* (%)	All*N* = 1034	Sex		Age	
		Male*N* = 730	Female*N* = 304	<65 years*N* = 329	≥65 years*N* = 705
Level of discomfort (VAS* score)					
None (1)	343 (33.2)	233 (31.9)	110 (36.2)	61 (18.5)	282 (40.0)
Mild (2–4)	498 (48.2)	356 (48.8)	142 (46.7)	186 (56.5)	312 (44.3)
Moderate (5–7)	161 (15.6)	120 (16.4)	41 (13.5)	65 (19.8)	96 (13.6)
Severe (8–10)	32 (3.1)	21 (2.0)	11 (3.6)	17 (5.2)	15 (2.1)

*n*, number reporting; *N*, total number of evaluable eFCs for assessment.

* Scale of 1–10 where 1 = no discomfort to 10 = extreme discomfort.

##### Discomfort by Qualitative Ranking

Across 1034 eFCs for which a VAS score was provided, the qualitative ranking indicated most eFCs caused mild discomfort (VAS = 2–4; 498/1034 [48.2%]) or no discomfort (343/1034 [33.2%]). Just 3.1% (32/1034) of eFCs were ranked as severe (VAS = 8–10), with the remaining 15.6% (161/1034) ranked as moderate (VAS = 5–7). The median (range) discomfort score was 2 (1–10).

The eFCs in patients aged ≥65 years were over twice as likely to be rated as causing no discomfort (≥65 years: 282/705 [40.0%]; <65 years: 61/329 [18.5%], *P* < 0.001).

Qualitative ranking of discomfort reported by males and females was similar to that for the overall analysis (*P* = 0.40). Most eFC were ranked mild (males: 356/730 [48.8%]; females: 142/304 [46.7%]) or no discomfort (males: 233/730 [31.9%]; females: 110/304 [36.2%]).

## Post‐eFC UTI

### Overall Results for UTI

For all eFCs, the post‐eFC UTI rate was 13.3% (142/1068), shown in Table 3.

**Table 3:** Summary of post‐eFC UTI rate – by sex within age subgroup.

Post‐eFC UTI, *n* (%)	Age <65 years			Age ≥65 years		
	All*N* = 339	Male*N* = 219	Female*N* = 120	All*N* = 729	Male*N* = 528	Female*N* = 201
Yes	30 (8.8)	14 (6.4)	16 (13.3)	112 (15.4)	70 (13.3)	42 (20.9)
No	309 (91.2)	205 (93.6)	104 (86.7)	617 (84.6)	458 (86.7)	159 (79.1)

*n*, number reporting; *N*, total number of evaluable eFCs for assessment.

The post‐eFC UTI rate in females was >50% higher than that observed in males (females: 58/321 [18.1%]; males: 84/747 [11.2%]; *P* = 0.003).

The eFCs conducted in older participants also had a higher UTI rate than younger participants (<65 years: 30/339 [8.8%]; ≥65 years: 112/729 [15.4%]; *P* = 0.004).

### Post‐eFC UTI by pre‐eFC dipstick analysis

For the 12 eFCs with positive dipsticks pre‐eFC, only 16.7% (two of 12) were associated with a post‐eFC UTI. This rate was similar to the rate when the pre‐eFC dipstick analysis was negative (103/720 [14.3%]).

The UTI rate where no pre‐eFC urine analysis was performed, and participants were asymptomatic for UTI was 11% (37/336).

### Relationship between tolerability and post‐eFC UTI

There was no statistical difference between discomfort ranking and post‐eFC UTI status (*P* = 0.399). The modal ranking for discomfort was ‘none’ for eFCs associated with a post‐eFC UTI and for those with no post‐eFC UTI (54/130 [41.5%] vs 437/881 [49.6%], respectively).

There were no recorded instances of urosepsis‐related hospital admission or mortality following eFC.

The most common indications for undergoing evaluation by eFC were:

- LUTS (312/1091 [28.6%])
- TCC surveillance (300/1091 [27.5%])
- Visible haematuria (167/1091 [15.3%])
- Recurrent UTIs (130/1091 [11.9%])
- ‘Other’ reasons (each accounted for <10% of all eFCs)

## Discussion

This study evaluated both the tolerability and safety of eFC for a range of symptoms and conditions within urology. This large prospective study across a wide age range, in both sexes with minimal exclusion criteria, allowed for robust and reliable analysis, representative of a broad, real‐world selection of patients.

### Discomfort

The study evaluated data from participants who underwent one or more eFCs; 1091 eFCs in 933 participants making it the largest prospective study of eFC to date. Overall, patients did not find eFC uncomfortable, a finding in line with other studies with significantly lower participant rates [13, 14, 15].

Despite a statistically significant difference observed for discomfort levels between those aged <65 and ≥65 years (*P* < 0.001), with those in the older age category reporting less discomfort, this is unlikely to be clinically relevant, as at least three‐quarters of participants in each subgroup rated eFC as either causing no, or mild discomfort. Within the age subgroups, VAS scores were similar between sexes.

Most patients do not find eFC unduly painful. In a study by Abed et al. [14] of 132 patients undergoing eFC, the mean VAS pain score was 2.55 (0 = no pain, 10 = worst pain), findings comparable to those in patients undergoing standard FC (no EndoSheath; mean VAS pain score 2.48 [0 = no pain, 10 = excruciating unbearable pain]) [7].

Burke et al. [19], reported that only 1.8% of patients undergoing standard FC assigned a mean VAS pain score of >5 (0 = no pain, 10 = worst pain), all of whom were male [19]. A difference in mean VAS pain scores between sexes was also observed by Greenstein et al. [7] (males = 2.8; females = 1.97; 0 = no pain, 10=excruciating unbearable pain).

Patients undergoing eFC are generally highly satisfied with the procedure. A previous study by Krebs et al. [15] of 97 patients found overall satisfaction was similar in eFC (*n* = 49) and no EndoSheath FC groups (*n* = 48) (*P* = 0.94), with patients willing to undergo a repeat eFC if clinically indicated [14].

### The UTI Incidence

The post‐eFC UTI rate at 13.3%, appears to be higher than the reported 2–7.5% in studies where no EndoSheath was used [2, 17, 19, 20]. However, there are several explanations for this mostly related to definition of UTI, duration of follow‐up, and study population.

Clark and Higgs [17], reported on a much smaller sample size (161 vs 1034 FCs). In addition, they classified a UTI as significant bacteriuria (>10^5^ colony‐forming units [CFU]/mL) whereas the present study had a lower threshold with no need for a positive urine culture and with either symptoms or evidence of antibiotic prescription post‐eFC being adequate for diagnosis. Furthermore, they only reported UTIs in the first 3 days post‐eFC [17], not the 1‐month period used in the present study.

In the study by McCombie et al. [20], whilst the MSU‐confirmed UTI rate was 2.0%, 13.3% of participants were given antibiotics based on new UTI symptoms, similar to the UTI rate observed in this study that used similar diagnostic criteria. This emphasises the fact that the MSU‐defined UTI rate is much lower than the rate of new urinary tract symptoms occurring after FC, which are often treated by GPs with antibiotics on the assumption that they are due to a UTI (but without a positive urine culture).

Cusumano et al. [2], in a study of 139 patients used the same UTI criteria as this study with a reported UTI rate of 4.3%. However, it was a retrospective study, and the goal sample size could not be achieved due to missing documentation related to antimicrobial administration prior to FC. In addition the study only included one female patient.

In contrast, Almallah et al. [21], reported that 11.6% (12/103) of participants had UTI symptoms post‐FC but only 4.8% (five of 103) had significant bacteriuria (>10^5^ CFU/mL), once again emphasising that the proportion of patients with significant bacteriuria is much lower than those with urinary symptoms after FC.

Antibiotic prophylaxis is not standard practice prior to FC but can be considered if the patient has a history of recurrent UTIs, abnormal anatomy, uses a catheter or is immunocompromised [21, 22]. This view is supported by the fact that the incidence of symptomatic UTIs in patients administered prophylactic antibiotics (2.2%) and in those who were not (2.5%), showed no statistical difference (*P* > 0.99) [2]. Whilst in another study where all patients received prophylactic antibiotics, 24% developed new UTI symptoms but none developed bacteriuria or a UTI [16], a finding corroborated by others [23].

The recently reported randomised control trial of antibiotics vs methenamine hippurate for the prevention of recurrent UTIs in women showed that only 48% of clinical UTIs were confirmed by a positive urine culture [24]. If these stricter criteria for UTI definition had been used in the present study, then, given the known rates of MSU testing, the UTI rate would have been <6.5% overall.

Thus, the UTI rate observed in the present evaluation is in line with the reports above and may be explained by the fact that an older population was studied, the duration of follow‐up at 30 days was longer, the study population unselected, and the definition of a UTI was not based on a positive MSU culture alone.

The post‐eFC UTI rate for participants who returned the second survey (UTI self‐reported) was 13.2% (39/296), similar to the UTI rate for participants whose UTI status was obtained from CHARTS 13.3% (103/772), indicating that diagnosis of UTI by either means was consistent and that pooling of UTI information from the two groups appropriate.

These results question whether a UTI diagnosis in general can be based solely on patient‐reporting of UTI symptoms, without considering significant bacteriuria as indicated by raised leucocytes and nitrites on dipstick urine analysis. In addition, asymptomatic bacteriuria can be especially common in the elderly population [25, 26] (most participants in this study). Therefore, a case can be made for abandoning pre‐eFC dipstick urine analysis in the absence of UTI symptoms on direct patient questioning, as a positive dipstick was seen in just 1% of such patients of whom only 16.7% went on to have treatment for a UTI, which was only slightly higher than when the pre‐eFC UTI assessment was negative (14.3%). It further supports the current guidance stating that routine prophylactic antibiotics for FC is not necessary.

Overall, this prospective study's uniquely large prospective sample suggests that the perceived UTI rate post‐eFC is close to 13%. Given the significant variation in defining a UTI and in the post‐eFC follow‐up times, it is difficult to compare the UTI rates observed in this study with those in the published literature, but where inclusion criteria are similar so are the rates of UTI.

As UTI rates are generally low, it appears intuitive to wait for a patient to present to their GP if they have symptoms indicative of a UTI and then prescribe antibiotics accordingly. Herr [16] (2014), stressed the importance of antibiotic stewardship after cystoscopy. This is particularly important when high proportions of patients report possible UTI symptoms but do not have significant bacteriuria in the short period after FC, and do not later develop a UTI.

Interestingly, this study noted there was no relationship between procedural discomfort and the presence of post‐eFC UTI (i.e., increased discomfort did not result in an increased likelihood of developing a post‐eFC UTI (*P* = 0.399).

Some limitations of the study include the lack of control group, and missing data in the evaluable data set. However, the missing data rate was low (4–5%) and given the numbers studied both acceptable and unlikely to affect the study conclusions.

In the study design patients were not required to provide a pre‐eFC urine specimen if they were asymptomatic for a UTI and unable to provide a sample. This approach is supported by research by Trail et al. [27] where they found that patients with a positive pre‐FC urine dipstick did not go on to develop post‐FC urinary sepsis, an outcome supported by Chavarriaga et al. [28]. Another limitation is that patients were not routinely followed up post‐eFC with a urine dip or MSU sample as funding was not available for this. As previously discussed, there are high rates of asymptomatic bacteriuria especially in older patients and therefore patient self‐reporting of symptoms suggestive of UTI were determined as being positive for a UTI. However, infection rates were low and there were no cases of urosepsis.

### Costs and Sustainability

The per use estimated cost for eFC is £92.41 to £137.61 and that for a disposable cystoscope is £150 (see comment responses for calculations). The EndoSheath costs £41.45 whereas the reprocessing costs and sterilisation for a reusable cystoscope are estimated to be £107 per scope [29], with reusable scopes becoming more economical than single use scopes once ≥200 procedures are performed [29]. These data further supports EndoSheath use.

Kemble et al. [30] reported that the carbon footprint of a single use scope at 2.4 kg CO_2_ is significantly more than for a reusable scope (0.53 kg CO_2_). Whilst the paper does not specifically mention eFC, its carbon footprint will be similar to that for a reusable cystoscope the main difference being that whilst there is no reprocessing required for eFC use (saving 0.20 kg CO_2_) the EndoSheath (60 g, polyurethane) has to be manufactured and recycled, with an estimated carbon footprint for this of 0.21 kg CO_2_.

### Personal Observations

In practical terms the author (B.B.), having used all three types of cystoscopes, would comment that for general purpose use there is little to choose between them. The reusable scope is slightly smoother to use in practice than either of its counterparts but otherwise no differences are appreciated.

## Conclusion

An eFC is a well‐tolerated and safe procedure, with the potential for significant savings in time and therefore cost and should be considered for wider use.

## Disclosure of Interests

Laborie contributed €500 to aid recording of a presentation to the International Continence Society but had no input into the design, protocol or analysis of the submitted study, which was completed before contact with Laborie was made and the funding was offered.

## Supporting information


**Data S1** FLEXI CYSTOSCOPY PATIENT QUESTIONNAIRE: POST PROCEDURE INFECTION.
