# Supplementary figures and images for: EndoSheath use in flexible cystoscopy: a prospective evaluation of >1000 cases
Source: BJU Int. 2024 Nov 12;135(3):489–96. doi: 10.1111/bju.16578 (PMC11842883; doi:10.1111/bju.16578)

Supplementary material:


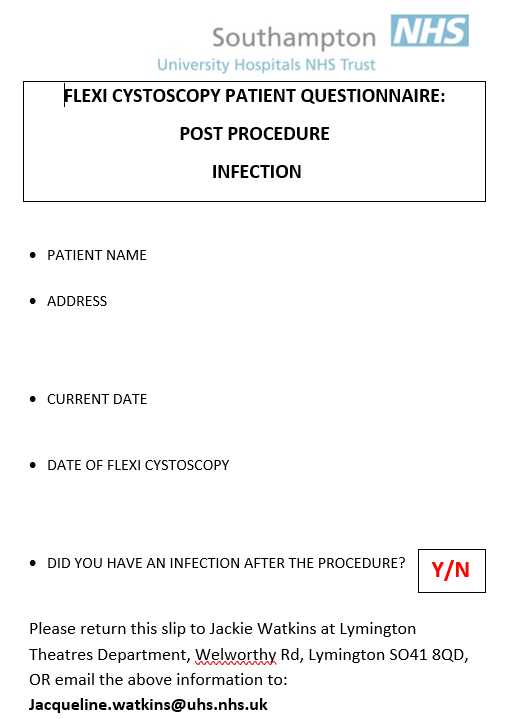


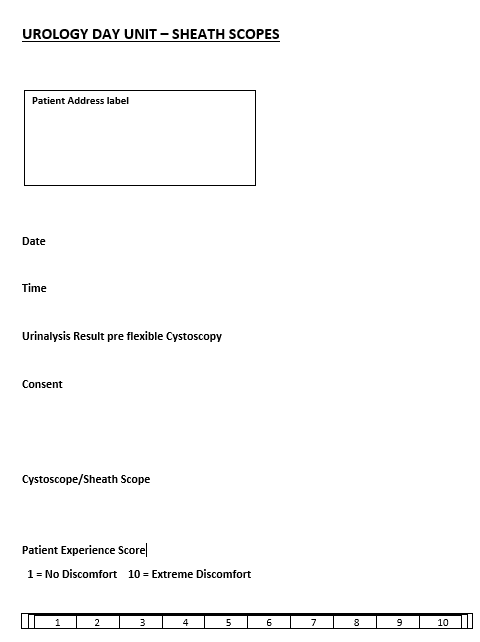

Supplement: Supplementary file 1 — Data S1 FLEXI CYSTOSCOPY PATIENT QUESTIONNAIRE: POST PROCEDURE INFECTION. [file BJU-135-489-s001.docx]
